# First Genome Sequence of Pasteurella multocida Type B Strain BAUTB2, a Major Pathogen Responsible for Mortality of Bovines in Bangladesh

**DOI:** 10.1128/MRA.00901-18

**Published:** 2018-09-06

**Authors:** M. Shahjahan Ali Sarker, M. Tanvir Rahman, M. Muket Mahmud, Massimiliano S. Tagliamonte, Shah M. Ziqrul Haq Chowdhury, Mohammad Rahfiqul Islam, M. Bahanur Rahman, Mohamed E. El Zowalaty, K. H. M. Nazmul Hussain Nazir

**Affiliations:** a Department of Microbiology and Hygiene, Faculty of Veterinary Science, Bangladesh Agricultural University, Mymensingh, Bangladesh; b Department of Pathology, College of Medicine, University of Florida, Gainesville, Florida, USA; c Emerging Pathogens Institute, University of Florida, Gainesville, Florida, USA; d Livestock Division, Bangladesh Agricultural Research Council (BARC), Farmgate, Dhaka, Bangladesh; e Virology and Microbiology Research Laboratory, School of Health Sciences, College of Health Sciences, University of KwaZulu-Natal, Durban, South Africa; Loyola University Chicago

## Abstract

Here, we report the first genome sequence of Pasteurella multocida BAUTB2 isolated from a buffalo that died from hemorrhagic septicemia in Rajshahi, Bangladesh. Using Illumina HiSeq technology, the BAUTB2 genome length was determined to be 2,439,149 bp, with 40.8% GC content, 2,307 coding sequences (CDS), 6 rRNAs, 51 tRNAs, and 4 noncoding RNAs (ncRNAs).

## ANNOUNCEMENT

It has been over 135 years since Louis Pasteur showed that Pasteurella multocida is the causative agent of fowl cholera (1). P. multocida is the etiological agent of hemorrhagic septicemia, an acute fatal septicemic disease of cattle, buffalo, and other large ruminants. P. multocida is a Gram-negative, nonmotile, rod-shaped, and economically important opportunistic pathogen causing multihost diseases manifested by pneumonic pasteurellosis and hemorrhagic septicemia in bovine, atrophic rhinitis and pneumonic pasteurellosis in swine, fowl cholera in avian species, hemorrhagic septicemia in cattle and rabbit, acute and chronic pneumonia in ovine species and camelid, and bite, respiratory, and a range of zoonotic infections in humans (2–5). Serologically, P. multocida strains are classified into five serogroups (A, B, D, E, and F) based on K antigens, whereas, according to the O antigens, P. multocida strains are classified into 16 somatic serotypes (6).

Characterization of the complete genome of Pasteurella is of great interest due to its clinical and veterinary importance. It allows further in-depth analysis of the genomic structure and provides insights about comparative pathogenomic characteristics of the bacterium. Further analysis will help identify potential targets with a genetic basis for development of rapid accurate diagnostics and vaccines to prevent and control the diseases in animal populations. The genome sequence of P. multocida BAUTB2 is the first genome sequence of a P. multocida strain from Bangladesh to be published.

Here, we announce the draft genome sequence of P. multocida BAUTB2 isolated in April 2016 from a buffalo that died from hemorrhagic septicemia in Rajshahi, Bangladesh. The isolate was initially cultured, isolated on blood agar medium, and identified based on conventional biochemical characteristics. The isolate was confirmed as type B based on molecular methods of PCR using type-specific primers targeting the capsular gene, as was previously described (7, 8). For genome sequencing, DNA was isolated using a Wizard genomic DNA kit (Promega Corp., Madison, WI), according to the manufacturer’s instructions. Genome sequencing of the strain was performed by Genewiz (Suzhou, China) using the Illumina HiSeq technology (1,151,332 trimmed reads, 109-fold mean coverage). Sequences were assembled using SPAdes v.3.11.0 (9) into 157 contigs at least 250 nucleotides long for a total of 2,439,149 bp. Annotation was performed using the Rapid Annotations using Subsystem Technology (RAST) server (9–11).

**FIG 1:**
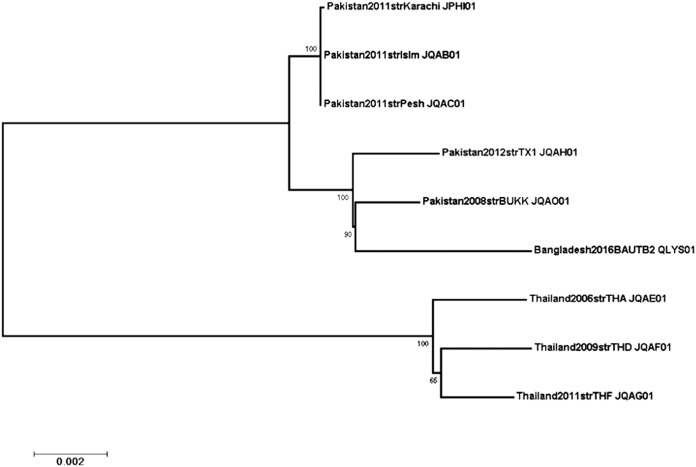
Maximum likelihood phylogeny of P. multocida BAUTB2 strain from Bangladesh. Phylogenetic tree was constructed using variable regions from the whole-genome alignment of strain BAUTB2 with P. multocida B:2 assemblies from Southeast Asia. The maximum likelihood tree was calculated with IQ-Tree v.1.5.5 (17, 18) using the model K3P+ASC, with 1,000 bootstrap replicates (19). Branch labels indicate bootstrap percent support. The closest relative to BAUTB2 is the Pakistani strain BUKK (WGS Project, JQAO01; GenBank assembly accession number GCA_001029495).

For phylogenetic analysis, 49 contigs with 500-nucleotide minimum lengths were aligned to 8 other assemblies of P. multocida B:2 strains (NCBI WGS project numbers JQAF01, JQAE01, JPHI01, JQAH01, JQAG01, JQAO01, JQAC01, and JQAB01) using progressiveMauve v.2.4.0 (12). Variant loci from conserved segments of at least 500 nucleotides long were extracted, and this alignment subset was assessed for recombination using GARD (13) in the HyPhy v.2.3 package (14). The largest partition included 255 variant sites, which were used to calculate a maximum likelihood tree (15, 16) using IQ-TREE v.1.5.5 (17, 18) with 1,000 bootstrap replicates (19). As shown in Fig. 1, the strain BAUTB2 was clustered with the Pakistani strain (GenBank assembly accession number GCA_001029495) with high bootstrap support.

### Data availability.

This whole-genome shotgun project has been deposited in DDBJ/ENA/GenBank under the accession number QLYS00000000. The version described in this paper is the first version, QLYS01000000.
